# Liquid Chromatography-Mass Spectrometry Metabolomic Analysis of *Terminalia ferdinandiana* Exell. Fruit Extracts That Inhibit HIV-1 Cell Infection, HIV-1 Reverse Transcriptase and HIV-1 Protease

**DOI:** 10.3390/molecules30081701

**Published:** 2025-04-10

**Authors:** Ian Edwin Cock, Benjamin Matthews, Adriaan Erasmus Basson

**Affiliations:** 1 Centre for Planetary Health and Food Security, Griffith University, Brisbane 4222, Australia; 2 Queensland Brain Institute, University of Queensland, Brisbane 4072, Australia; benjamin.matthews@uq.edu.au; 3 Department of Molecular Medicine and Haematology, School of Pathology, Faculty of Health Sciences, University of the Witwatersrand, Parktown, Johannesburg 2193, South Africa; adriaan.basson@wits.ac.za

**Keywords:** *Combretaceae*, *Kakadu plum*, HIV-1 replication cycle, viral cell entry assay, HIV-1 reverse transcriptase, HIV-1 protease, metabolomics profiling, tannin, flavonoid, stilbene

## Abstract

The emergence of HIV strains resistant to the current anti-retroviral drugs has necessitated the search for new anti-retroviral medications. Methanolic and aqueous *T. ferdinandiana* fruit extracts have potent inhibitory activity against several phases of the HIV-1 replicative cycle. Cell infectivity studies using a non-resistant HIV-1 pseudovirus demonstrated that the methanolic (IC_50_ 16 µg/mL) and aqueous extracts (IC_50_ 19 µg/mL) were potent inhibitors of viral infection in a non-replicating HIV-1 assay. Both extracts also inhibited HIV-1 reverse transcriptase (IC_50_ values of 35 and 33 µg/mL for methanolic and aqueous extracts, respectively) and HIV-1 protease (IC_50_ values of 19 and 27 µg/mL, respectively) in recombinant enzyme assays. Given their inhibitory activities against multiple phases of HIV-1 replication, *T. ferdinandiana* fruit extracts may be particularly useful as HIV-1 therapeutics. Furthermore, both extracts displayed good safety profiles and therapeutic indices, indicating their suitability for therapeutic usage. LC-MS metabolomic profiling analysis of the methanolic extract identified several interesting constituents, including a relative abundance of tannins, as well as several flavonoids and stilbenes. All of these compounds have previously been reported to have bioactivities consistent with the anti-HIV-1 activities reported herein. Based on these studies, methanolic and aqueous *T. ferdinandiana* fruit extracts are promising potential therapies for the prevention, treatment and management of HIV-1.

## 1. Introduction

One of the most daunting and difficult-to-treat diseases globally is the current HIV/AIDS pandemic. Since its first detection in the early 1980s, HIV has generated considerable fear due to the fact that it is currently non-curable and is generally fatal without treatment. Due largely to public education campaigns in the 1980s and 1990s, the spread of the disease has been limited with some success in several regions of the world (USA, Europe, Australia), although it has continued to steadily increase in other regions (including several regions of Africa and Asia) [1]. There is recent concern over the increase in newly diagnosed cases of HIV, with levels in developed countries increasing substantially in recent years. For example, the rates of new HIV infections in Australia nearly doubled in the period between 2010 and 2012 and have remained at this increased rate of infection since then [1,2]. The greatest increase in the diagnosis of new cases is for those aged under 25. The Australian figures are also mirrored by European and American figures, which also show recent increases in newly diagnosed HIV/AIDS cases, especially in those under 25 years of age [1].

The current management regime for HIV is to provide the infected individual with anti-retroviral drugs in an attempt to control the infection. The most commonly used drugs for HIV/AIDS are reverse transcriptase inhibitors (particularly nucleotide analogues, which block viral RNA being transcribed into a DNA copy). Other anti-retroviral drugs may target viral entry into the host cell or the incorporation of viral DNA into the host DNA. Further anti-retroviral drugs target an HIV protease enzyme, which blocks viral budding after replication via inhibiting mature viral protein production. All of these treatments affect the viral replication cycle but are generally only partially effective on their own. For this reason, combinations of anti-retroviral drugs, often targeting different phases of the viral replication cycle, may be prescribed for more effective management of HIV/AIDS [3]. Not only are combinations such as these more potent and provide superior therapeutic outcomes compared with standard HIV monotherapies, but they also inhibit the development of further HIV-1 resistance mechanisms by substantially decreasing HIV replication rates. However, none of the current treatments are completely effective, and resistance to all currently approved anti-retroviral drugs has been reported [4,5,6]. One of the most commonly used anti-retroviral drugs, azidothymidine (AZT, also known as zidovudine), functions as an inhibitor of HIV reverse transcription by competing with normal nucleotides for incorporation into viral DNA. There have been recent reports that some strains of the most prevalent HIV virus (HIV-1) have mutated their reverse transcriptase enzyme to provide it with a proofreading function [4]. This essentially makes AZT of little or no use in the treatment of these strains of HIV-1. Similarly, prolonged and incorrect usage of individual anti-retroviral drugs has resulted in new viral strains that are resistant to some or all of the currently used treatments. There is a very real need to develop new treatments for HIV/AIDS.

Traditional therapies and herbal medicines are good candidates for the development of new remedies against pathogenic disease, including viral pathogens. In many cases, their use is well documented, assisting with species selection. Furthermore, plant-based remedies consist of multiple components, may have several beneficial bioactivities and therefore may potentially function as combinational therapies. Plants of the genus *Terminalia* are especially promising and are amongst the most widely used plants globally in traditional medicines [7]. In general, *Terminalia* spp. have high contents of antioxidant compounds. Additionally, they are also characterised by their high levels of flavonoids and tannins. It has been postulated that these classes of molecules may contribute (at least in part) to medicinal bioactivities of *Terminalia* spp. [7]. Several species have been reported to have antiviral properties. *Terminalia chebula* Retz. extracts are good inhibitors of measles, mumps [8] and herpes simplex viruses (HSV-1) [9]. Notably, several *Terminalia* spp. also inhibit HIV replication. The South American species *Terminalia triflora* (Griseb.) Lillo blocks HIV infections by inhibiting reverse transcriptase enzyme activity [10]. Similarly, the African species *Terminalia mollis* M.A.Lawson also inhibits HIV-1 replication [11], although that study did not examine the anti-HIV mechanisms of the extracts. The Australian species *Terminalia ferdinandiana* Exell. has been reported to have potent inhibitory activity against bacterial [12] and protozoal parasites [13]. Despite these earlier studies, *T. ferdinandiana* is yet to be examined for inhibitory activity against viral replication. This study investigated the effects of *T. ferdinandiana* fruit extracts against three phases of the HIV-1 replicative cycle.

## 2. Results

### 2.1. Rapid Screen for Antiviral Activity: Inhibition of MS2 Phage Replication

**Figure 1:**
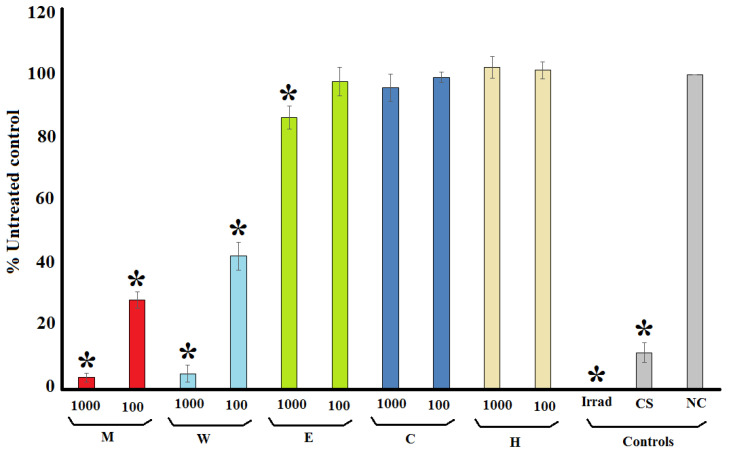
MS2 plaque formation in an F+ Amp+ *E. coli* lawn, expressed as % of the untreated control plaque formation following incubation of the MS2 bacteriophage with the *T. ferdinandiana* fruit extracts at 1000 and 100 µg/mL. M = methanolic extract; W = deionised water extract; E = ethyl acetate extract; C = chloroform extract; H = hexane extract; Irrad = microwave irradiation (positive control); CS = *Camellia sinensis* leaf water extract (1000 µg/mL; positive control); NC = nutrient broth (negative control). All results are reported as the mean of three assays performed in triplicate ± SEM (*n* = 9). * indicates statistically significant results (*p* < 0.01).

*Terminalia ferdinandiana* extracts were prepared using solvents of varying polarities and were initially screened in the MS2 phage plaque reduction assay to narrow the focus for further studies. The MS2 phage assay was selected for these initial screening studies as it has similar replicative cycles as several medically important human RNA viruses and has previously been reported to be a relevant model for rapid screening [14]. Figure 1 shows the change in formation of MS2 bacteriophage plaques in the presence of the extracts or the positive and negative controls as a % of the untreated control plaque formation. The methanolic and aqueous *T. ferdinandiana* extracts were good inhibitors of MS2 plaque replication. Indeed, both extracts inhibited >95% of viral replication (as determined by decrease in plaque reduction) at 1000 μg/mL, and between 58 and 72% reduction at 100 μg/mL for the aqueous and methanolic extracts, respectively. The inhibitory activity of these extracts was further quantified by testing across a range of concentrations and the efficacy was expressed as the concentration required for a 50% reduction in plaque formation (IC_50_). Both extracts were good MS2 replication inhibitors, with IC_50_ values of 68 and 44 μg/mL for the methanolic and aqueous extracts, respectively (Table 1). Exposure to the ethyl acetate extract also induced a reduction in plaque formation. However, the decrease was substantially less apparent than for the methanolic and aqueous extracts, with a 14% reduction in plaque formation recorded at 1000 μg/mL and no reduction evident at 100 μg/mL. The chloroform and hexane extracts completely lacked anti-MS2 phage activity, with no significant reduction compared to the untreated control noted at either test concentration. Thus, only the methanolic and aqueous extracts were deemed suitable for further examination by specific HIV-1 assay systems.

### 2.2. Inhibition of HIV-1 Reverse Transcriptase

**Figure 2:**
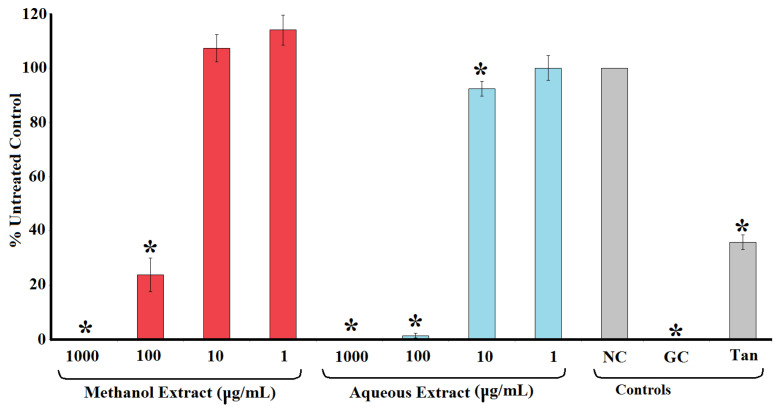
HIV-1 reverse transcriptase activity following exposure of the recombinant enzyme to varying concentrations of methanolic and aqueous *T. ferdinandiana* fruit extracts. The results are expressed as % of the untreated control. Tannic acid (10 μm) was included as a positive control. NC = untreated control; GC = growth control; Tan = tannic acid control. All results are reported as the mean of three independent assays performed in triplicate ± SEM (*n* = 9). * indicates results that are significantly lower than the untreated control (*p* < 0.01).

Figure 2 shows the inhibition of HIV-1 reverse transcriptase activity following exposure to methanolic and aqueous *T. ferdinandiana* extracts. Both extracts were strong inhibitors of the recombinant HIV-1 reverse transcriptase enzyme. Indeed, the enzyme was completely inhibited when exposed to 1000 μg/mL of either extract (Figure 2). Notably, these extracts were better inhibitors than the tannic acid control (10 μm), which inhibited HIV-1 reverse transcriptase by approximately 60%. Similarly, the 100 μg/mL concentration of both extracts also strongly inhibited HIV-1 reverse transcriptase, with activity reductions of approximately 77% and 99% for the methanolic and aqueous extracts, respectively. The potency of enzyme inhibition was further quantified, with IC_50_ values of 35 and 33 μg/mL calculated for the methanolic and aqueous *T. ferdinandiana* fruit extracts, respectively (Table 1).

### 2.3. Inhibition of HIV-1 Protease

**Figure 3:**
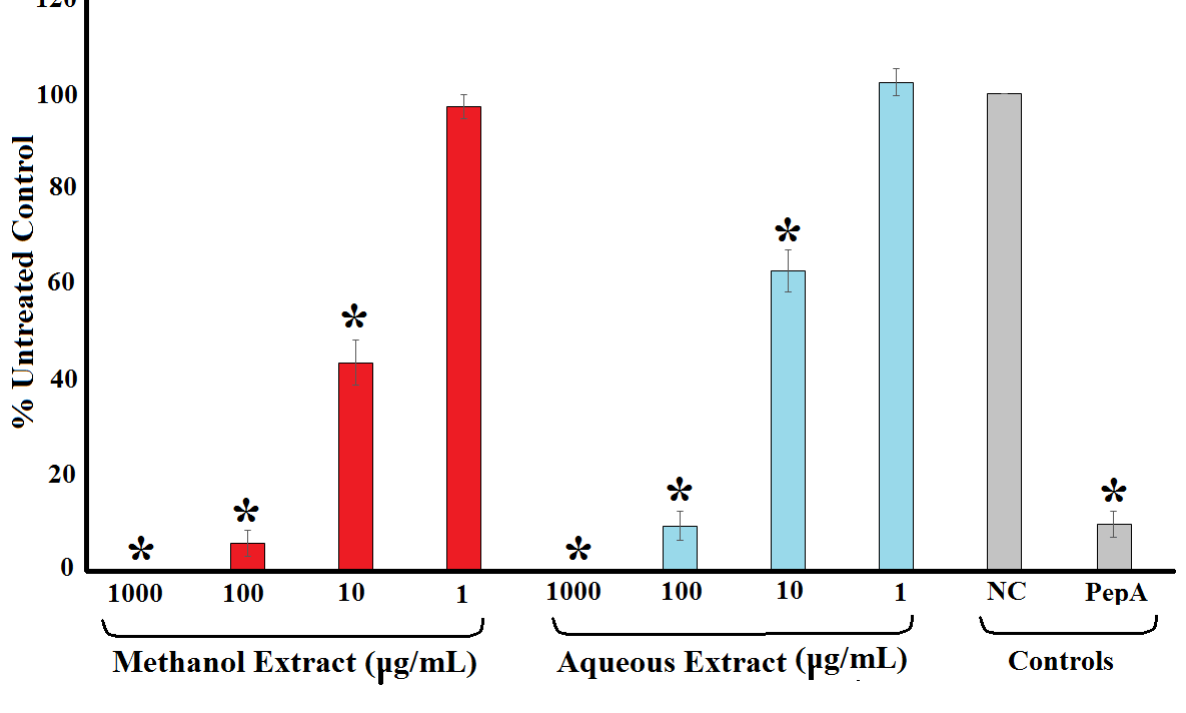
HIV-1 protease activity reduction following exposure to varying concentrations of methanolic and aqueous *T. ferdinandiana* fruit extracts expressed as % of the untreated control. Pepstatin A (10 μm) was included as a positive control. NC = untreated control; PepA = pepstatin A. All results are reported as the mean of three independent assays performed in triplicate ± SEM (*n* = 9). * indicates results that are significantly lower than the negative control (*p* < 0.01).

The ability of the methanolic and aqueous *T. ferdinandiana* extracts to inhibit HIV-1 protease activity was evaluated using a synthetic peptide substrate (Arg-Glu(EDANS)-Ser-Gln-Asn-Tyr-Pro-Ile-Val-Gln-Lys(DABCYL)-Arg), which fluoresces at 490 nm when it has been cut after the tyrosine residue. Both the methanolic and aqueous extracts were strong inhibitors of the HIV-1 recombinant protease enzyme. Indeed, the protease activity was completely inhibited when exposed to 1000 μg/mL of either extract (Figure 3). Similarly, 100 μg/mL of both extracts inhibited HIV-1 protease activity by >90%. Notably, protease inhibition was stronger for both extracts at 100 μg/mL than recorded for the positive control pepstatin A (10 μm). The protease inhibition by the extracts was concentration dependent, with the level of inhibition decreasing with decreasing extract concentration. Exposure to 10 μg/mL of the methanolic and aqueous extracts inhibited the enzyme’s activity by 57 and 38%, respectively, whilst the enzyme activity was not significantly different to the untreated control at 1 μg/mL. The potency of enzyme inhibition was quantified by IC_50_ determination, with IC_50_ values of 19 and 27 μg/mL determined for the methanolic and aqueous extracts, respectively (Table 1).

### 2.4. Inhibition of HIV-1 Cell Infection

**Figure 4:**
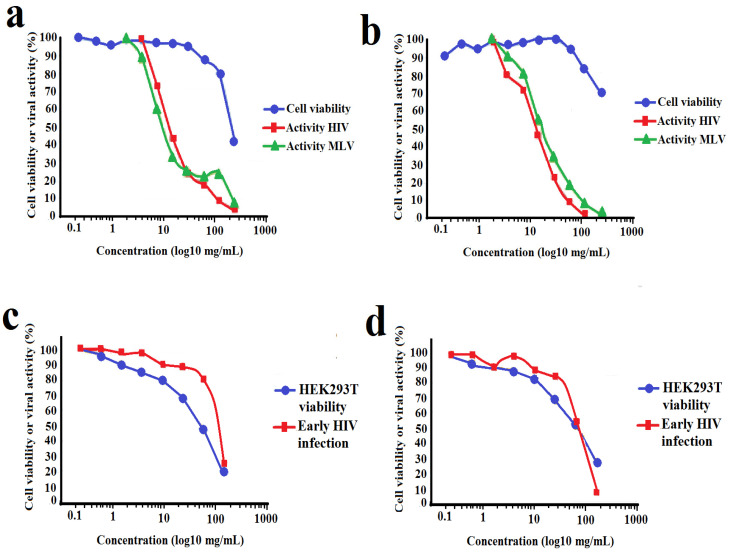
Cellular toxicity and inhibition of HIV-1 and MLV envelope-bearing pseudovirus entry into TZM-bl cells when exposed to varying concentrations of (**a**) methanolic and (**b**) aqueous *T. ferdinandiana* fruit extracts. Panels (**c**,**d**) show the effect of the methanolic and aqueous extracts, respectively, on early viral replication processes following cell entry. All results are expressed as % of untreated control cells and are reported as the mean of two independent assays performed in duplicate ± SEM (*n* = 4).

*Terminalia ferdinandiana* fruit methanol and water extracts strongly inhibited infection of TZM-bl cells by pseudoviruses (Figure 4a,b, Table 1). Both extracts had similar potencies against the HIV-1 enveloped pseudovirus, with IC_50_ values of 15.5 μg/mL and 19.2 μg/mL for the methanolic and aqueous extracts, respectively (Table 1). Interestingly, both extracts were similarly effective against the MLV enveloped pseudovirus, with IC_50_ values of 8.8 μg/mL and 18.5 μg/mL for the methanolic and aqueous extracts, respectively. Inhibition of cell entry by both the HIV-1 and MLV envelope-bearing pseudoviruses may be indicative of non-specific binding of extract components to the cell surface receptors and/or envelope proteins. The viability of the TZM-bl cells was also examined in parallel with the viral assays. The extracts proved to be of relatively low toxicity (IC_50_ ≥ 227 μg/mL) and did not induce substantial decreases in cell viability, except when exposed to the top concentration tested. For the inhibition of early HIV-1 replication (Figure 4c,d), a decrease in viral activity was observed to overlap with the decrease in cell viability due to toxicity of the extracts. As such, the decrease in luciferase reporter expression was due to toxicity to the HEK293T cells and neither of the extracts showed any significant inhibition of early HIV infection in this assay.

### 2.5. Quantification of Toxicity

The toxicity of the *T. ferdinandiana* fruit extracts was evaluated using two methods. The extracts were initially screened by *Artemia* lethality assay (ALA) for rapid preliminary toxicity screening across a range of concentrations, and the LC_50_ was calculated. For comparison, the reference toxin potassium dichromate (1000 µg/mL) was also tested in the bioassay. Plant extracts with LC_50_ values <1000 µg/mL following 24 h incubation have previously been defined as toxic [15]. Therefore, the methanolic and aqueous extracts were deemed to be of low toxicity, with LC_50_ values of 875 and 983 µg/mL, respectively. It is noteworthy that the toxicity detected in the ALA generally correlates with extracts that have previously been reported to have high antioxidant capacities [16]. Indeed, fruit methanol and aqueous extracts prepared in a similar manner were reported in that study to contain 600 and 263 mg ascorbic acid equivalents, respectively. The high antioxidant content of *T. ferdinandiana* fruit is largely due to high levels of ascorbic acid [17]. Although the *A. franciscana* nauplii lethality assay is generally quite robust, the nauplii are relatively sensitive to changes in pH [18,19]. Acidic pH can suppress the rate of mitochondrial protein synthesis and potentially be fatal to the nauplii. Several studies have demonstrated that high iascorbic acid contents in extracts can result in overestimations of toxicity [12]. Therefore, the ALA toxicity assay may result in incorrect determinations of the toxicity associated with such extracts. Instead, mammalian cell proliferation and/or cell viability assays may give a more accurate evaluation of the toxicity of extracts with high antioxidant capacities. Notably, >50% of human primary dermal fibroblast cells exposed to either the *T. ferdinandiana* methanolic or aqueous fruit extracts were viable (compared to the untreated control), indicating that these extracts are non-toxic.

### 2.6. HPLC-MS QTOF Analysis

**Figure 5:**
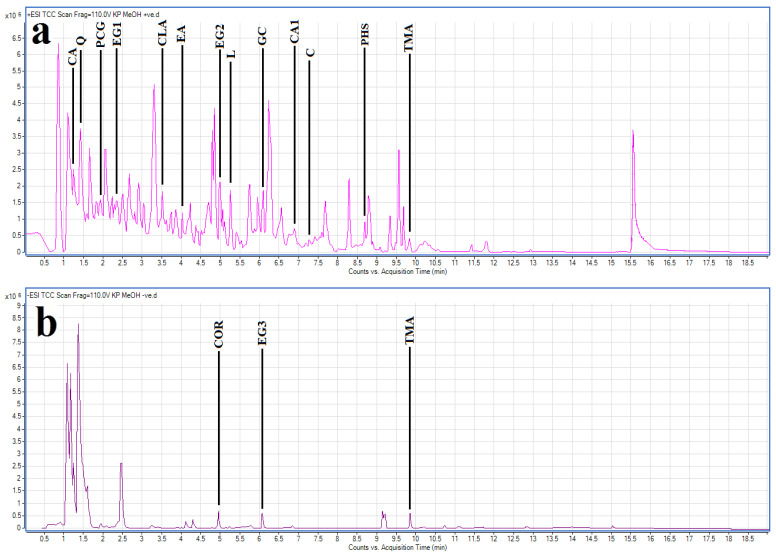
LC-MS chromatograms of methanolic *T. ferdinandiana* fruit extract in (**a**) positive and (**b**) negative ionisation modes with some noteworthy compounds highlighted. CA = chebulic acid; CLA = chlorogenic acid; C = combretastatin; CA1 = combretastatin A1; COR = corilagen; EA = ellagic acid; EG1, EG2, EG3 = ethyl gallate isomers; GC = gallocatechin; L = luteolin; PHS = pentahydroxystilbene; PCG = pyrocatechol glucuronide; TMA = trimethyl ellagic acid; Q = quercetin.

The methanol extract was selected for LC-MS metabolomic profile analysis, as compounds with a wider range of polarities are expected to be present compared with the aqueous extract. Optimised HPLC-MS parameters were developed and used to profile the methanolic *T. ferdinandiana* fruit extract. The resultant chromatograms are shown in Figure 5. In the methanolic *T. ferdinandiana* leaf extract, 561 unique molecular mass compounds were detected (unpublished results). Empirical molecular formulas were determined for each of those compounds using the QTOF analytical software (v4.3.0). Notably, we were able to putatively identify 443 of those unique mass signals against (79% of the total number) by comparing them to standards in two accurate mass databases, which comprised a unique plant compound database specifically generated for this study (800 compounds), as well as the Metlin metabolomics database, which consists of data on 24,768 compounds. A number of notable compounds were identified in the methanolic extract. Tannins were particularly abundant. In particular, chebulic acid, corilagen, ellagic acid, ethyl gallate isomers, gallocatechin, pyrogcatechol glucuronide and trimethyl ellagic acid were identified in the extract. The stilbenes combretastatin, combretastatin A1 and pentahydroxystilbene were also identified in the extract. Chlorogenic acid, and the flavonoids luteolin and quercetin, were also present in appreciable quantities in the extract. The most noteworthy compounds are summarised in Table 2.

## 3. Discussion

**Figure 6:**
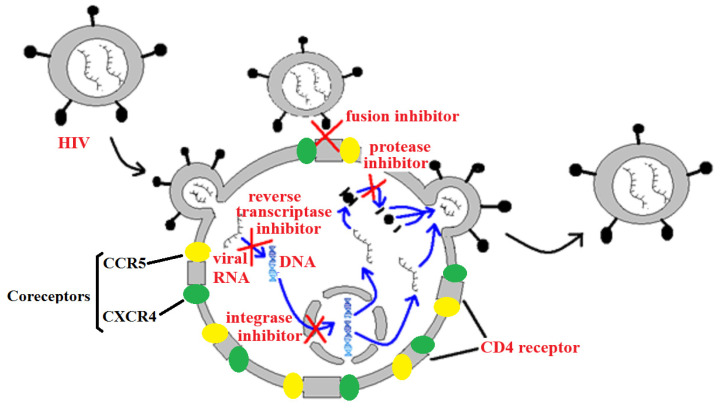
A representation of HIV viral replication, highlighting some targets for anti-retroviral chemotherapies.

Anti-retroviral therapies (ARTs) to treat HIV infection may target a number of phases of the HIV replicative cycle (Figure 6). As of August 2019, the FDA had approved thirty antiretroviral drugs and fifty-five drug formulations for the management and treatment of HIV-1 in the USA. The first of these drugs was the nucleoside reverse transcriptase inhibitor zidovudine (AZT). However, the HIV-1 reverse transcriptase enzyme has since developed a proofreading function, rendering AZT relatively ineffective in some drug-resistant HIV-1 strains [4]. The majority of the drugs currently approved by the FDA also target the reverse transcription process, accounting for more than half of the approved single entity drugs. Thirteen nucleoside/nucleotide reverse transcriptase inhibitors (NRTIs) and six non-nucleoside reverse transcriptase inhibitors (NNRTIs) are currently available clinically. A further nine anti-retroviral drugs approved by the FDA are HIV protease inhibitors, and four drugs inhibit HIV integrase. Inhibition of the HIV cellular infection processes are currently poorly represented, with only a single approved drug targeting viral fusion to the cellular CD4 receptor, and a further drug targeting the CCR5 co-receptor. However, the rapid emergence of resistant HIV strains has rendered some of these therapies of substantially lower efficacy. There is an urgent need to develop new anti-HIV therapeutic agents.

The management of HIV currently uses combinations of anti-retroviral drugs to increase efficacy and lessen the development of resistant strains [3]. These therapies typically consist of at least two nucleoside reverse transcriptase inhibitors and one non-nucleoside reverse transcriptase inhibitor. Alternatively, HIV combinational therapies may contain an HIV protease or integrase inhibitor in place of one of the reverse transcriptase inhibitors, and/or combinations of the latter two. The *T. ferdinandiana* extracts examined in this study inhibited several stages of the HIV replicative cycle and therefore also function as combinational therapies in their own right. HIV-1 reverse transcriptase activity was significantly inhibited by the methanolic and aqueous *T. ferdinandiana* extracts, with IC_50_ values of 35 and 33 µg/mL, respectively. Whilst our study demonstrated that the extracts inhibit HIV-1 reverse transcriptase activity, the inhibitory mechanism was not elucidated. However, it is likely that the tannin and flavonoid components may contribute to this inhibition. Interestingly, a diversity of tannins and flavonoids were detected in relatively high abundances in the methanolic *T. ferdinandiana* extract. Several of these have previously been reported to have anti-HIV-1 activities. Gallic acid is a potent inhibitor of HIV-1 reverse transcriptase, with an IC_50_ as low as 0.19 µg/mL, which compares well with the reference control nevirapine (1 µm) [20]. The tannins epigallocatechin (EGC) and epigallocatechin gallate (EGCG) are also potent inhibitors of HIV-1 reverse transcriptase activity, with IC_50_ values of 3.4 and 1.6 µm, respectively [21]. Both compounds also inhibit HIV-2 reverse transcriptase was similar efficacies. The same study also reported that both of these tannins inhibited the enzyme’s activity via allosteric inhibition and that the mechanism is different to all other NNRTIs approved for clinical use. Neither ECG nor EGCG bind to the NNRTI binding pocket but instead interact with a different region of the enzyme. The hydrolysable gallotannins shephagenins A and B also inhibit HIV-1 reverse transcriptase, with greater potency [22].

Flavonoids have also been reported to be good HIV-1 reverse transcriptase inhibitors. Quercetin (which was identified as a relatively abundant component in the *methanolic T. ferdinandiana* extract) has been reported to inhibit reverse transcription of RNA viruses [23]. Quercetin is also substantially less toxic than several reverse transcriptase inhibitors which are currently used clinically (arabinosides and acyclovir). However, whilst that study screened quercetin against reverse transcriptase activity, it did not use HIV-1 reverse transcriptase and activity against that enzyme needs to be confirmed. Of further note, flavonoids can also potentiate the reverse transcriptase inhibitory activity of some other reverse transcriptase inhibitors. For example, quercetin can substantially increase the reverse transcriptase activity of AZT when used in combination [24], confirming that different inhibitory mechanisms are used by the two compounds. Other flavonoids including baicalin [23] and xanthohumaol [25] also inhibit HIV-1 reverse transcriptase activity.

The *T. ferdinandiana* extracts were also potent inhibitors of HIV-1 protease activity, with IC_50_ values of 19 and 20 µg/mL, respectively. It is likely that the *T. ferdinandiana* tannins and flavonoids may also contribute to this inhibition as tannins have been reported to be particularly potent inhibitors of HIV-1 protease. Both ellagi- and gallotannins strongly inhibit HIV-1 protease activity in vitro [26]. The hydrolysable gallotannins corilagin and rapandusinic acid were particularly potent HIV-1 protease inhibitors, with IC_50_ values of 20 and 12 µm, respectively. Notably, corilagen was detected in relative abundance in the methanolic *T. ferdinandiana* extract in our study, and therefore it is likely to contribute to the decreased activity of this enzyme. The same study also examined inhibitory effects of 26 flavonoids on HIV-1 protease and reported inhibitory activity for the majority of the tested compounds. Quercetin was the most potent flavonoid inhibitor of HIV-1 protease activity (IC_50_ of 59 µm), whilst luteolin was a moderate protease inhibitor (IC_50_ not reported).

Both the methanolic and aqueous extracts were also good inhibitors of HIV-1 infectivity, with IC_50_ values of 16 and 19 μg/mL, respectively. This is particularly noteworthy and indicates that the extracts may inhibit cell-to-cell infection within an individual who has contracted the virus, thereby slowing the progression of the disease to AIDS. Equally interesting, the *T. ferdinandiana* extracts may also be useful in preventing new HIV-1 infections. Such a preventative application may be particularly useful for individuals in high-risk careers (e.g., medical professionals in emergency settings) or those with high-risk lifestyles (e.g., intravenous drug users, practitioners of unprotected sex, etc.). Whilst prophylactic administration of these extracts on their own would not guarantee protection against infection, it is likely that they would provide substantial protection by decreasing viral cell entry. However, our study used in vitro testing and does not take into account pharmacokinetic and pharmacodynamics effects which may modify the compounds or decrease their bioavailability in an in vivo system, and further testing is required.

Our study did not examine the mechanism(s) by which the *T. ferdinandiana* extracts inhibit HIV-1 infectivity. However, it is likely that several mechanisms may contribute to these effects. Compounds in the extracts may be interacting with either the cellular CD4 receptors or with the CCR5 co-receptor, thus decreasing the affinity of the receptor (or co-receptor) for the virus. Alternatively, the extract compounds may interact directly with the viral capsid. Interestingly, tannins are potent inhibitors of HIV-1-mediated cell fusion events by interfering with HIV-1 gp41 (a subunit of the HIV-1 envelope glycoprotein) and inducing conformational changes [27]. These changes substantially reduce fusion of the HIV-1 virion with the target cell, effectively blocking infection. That study tested five tannins and reported that all of them have potent anti-infective activity, with IC_50_ values ranging from approximately 5 to 20 µg/mL. Interestingly, LC-MS analysis of the methanolic *T. ferdinandiana* extract revealed tannins to be the most abundant and diverse class of compound present in the extract. It is likely that the *T. ferdinandiana* tannins may also inhibit the entry of HIV-1 into the cell by interfering with gp41. Interestingly, the *T. ferdinandiana* extracts also inhibited infection of an MLV envelope-bearing pseudovirus. This may indicate non-specific mechanisms are occurring to prevent viral cell entry. However, it is noteworthy that MLV contains a related envelope protein, gp71, which has similarities to the HIV-1 gp41 protein [28]. It is possible that the similar inhibitory mechanisms may block the entry of both viruses into the target cells.

Similarly, some flavonoids and stilbenes have been reported to inhibit HIV-1 infectivity. Several flavonoids decrease HIV-1 infection in vitro by inhibiting fusion of the virus with the cell membrane [23]. The flavonoids luteolin and quercetin were identified in the *T. ferdinandiana* methanolic extract in appreciable levels in our study, and they may also contribute to the inhibition of infection reported here. As previously noted [29], inhibition of infection by MLV envelope-bearing pseudoviruses in the TZM-bl assay may be indicative of inhibition of HIV-1 reverse transcriptase, and not entry per se. Indeed, we have shown in the enzyme-based assay that HIV-1 reverse transcriptase can be inhibited by both extracts. However, the cellular-based, early HIV-1 replication assay performed in this study, which is designed to detect inhibitors of HIV-1 reverse transcriptase, did not show any inhibitory activity by either of the extracts. A possible explanation for this observation could be the chemical properties of the active constituents. Tannin and flavonoid components (which were both detected in relative abundance in our study) are polar compounds and do not readily dissolve in the hydrophobic environment of cell membranes [30]. Furthermore, many tannins and some flavonoids (particularly flavonoid glycosides) are relatively large molecules. According to Lipinski’s rule-of-five model for the prediction of druggable compounds, molecules that have molecular masses >500 Da and/or the tendency to form hydrogen bonds are usually incapable of crossing lipid membranes by passive diffusion [31]. Passive transmembrane movement of many tannins and flavonoids alone is often very slow, and cells instead use active mechanisms to transport these compounds into the cell. For some of these transporters, intake of the phytochemical constituents requires symport co-transport of fructose [30]. Whilst the DMEM media used in our studies contains high glucose levels, it lacks added fructose, which may limit the ability of the HEK293T cells to transport flavonoids and tannins. Therefore, inhibition of HIV-1 reverse transcriptase may not be evident in these cells, whereas potent HIV-1 reverse transcriptase activity was evident in the cell-free enzyme inhibition assays. Alternatively, cellular efflux pumps may expel flavonoids and tannins as they enter the cell, thereby limiting their effects. Indeed, several ABC transporters (particularly ABCB1, ABCC1, ABCC2 and ABCG2) can expel flavonoids and tannins from cells, thereby lowering their intracellular concentrations and effects [30]. Interestingly, several of these ABC transporters have previously been reported in HEK293T cells [32], which may contribute to the low HIV-1 reverse transcriptase activity in the cellular assay compared to the enzyme-based assay. However, the inhibition of infection in the TZM-bl assay may therefore indicate pseudoviral cell entry as the major mechanism of inhibition (at least in that cell line).

Alternatively (or additionally), the *T. ferdinandiana* extracts may inhibit cellular infection via a further mechanism. A recent study reported that the cellular anion inositol hexaphosphate (IP6) (commonly known as phytic acid) stabilises the capsid proteins of the HIV-1 virion and facilitates viral uncoating [33]. That study demonstrated that interaction between IP6 and the viral capsid increases HIV-1 infection and the accumulation of viral DNA inside the cell by >100 fold. It is likely that any compound that modulates IP6 production or degradation may destabilise the HIV-1 capsid proteins and therefore decrease viral cell entry. Several tannins and flavonoids have been reported to inhibit several inositol phosphate kinases that catalyse phosphorylated inositol compounds, which are intermediates in IP6 synthesis [34]. That study identified several tannins including ellagic acid (IC_50_ 36 nm), epicatechin-3-gallate (94 nm) and epigallocatechin-3-gallate (EGCG; 120 nm) as particularly potent inhibitors of inositol phosphate kinases. The same study also identified several flavonoids including quercetin (180 nm) as good inhibitors of phosphorylated inositol products. Thus, these compounds inhibit IP6 synthesis, may decrease HIV-1 capsid stability and therefore result in decreased infection rates. Conversely, some stilbenes can potentiate the activity of some inositol phosphate phosphates, thereby stimulating the breakdown of the higher phosphorylated inositols, including IP6. One study reported that resveratrol (3,4′,5 trihydroxystilbene) stimulates the activity of an inositol polyphosphate phosphate, effectively dephosphorylating the higher phosphorylated inositols, thereby decreasing cellular IP6 levels [35]. The combined effects of the tannins on inositol phosphate kinases and the stilbenes on the inositol phosphate phosphatases may therefore result in decreased HIV-1 viral infectivity via destabilisation of the viral capsid proteins, although this is yet to be verified.

Our study did not examine whether the *T. ferdinandiana* extracts inhibit HIV-1 integrase activity. However, it is possible that future studies may also detect further inhibitory activities for the *T. ferdinandiana* extracts against this enzyme. Indeed, several flavonoids have been reported to inhibit HIV-1 integrase [36]. Similarly, chlorogenic acid (which was detected in relative abundance in the *T. ferdinandiana* methanolic extract) has been reported to have good HIV-1 integrase inhibitory activity [37]. Furthermore, whilst our study highlights multiple compounds that have previously been reported to have bioactivities consistent with the anti-HIV-1 activities reported herein, it is unlikely that any of these components is solely responsible for these properties. Instead, it is likely that the combination of the identified compounds may potentiate the activity of the individual compounds. Future studies are required to examine the effects of combinations of these compounds on HIV-1 cell entry, reverse transcriptase and protease activities. If potentiating combinations are identified, the optimal ratios of the compounds should also be determined to aid in the development of safe and effective HIV-1 therapies.

## 4. Materials and Methods

### 4.1. Materials

Unless otherwise stated, all reagents were obtained from Sigma, Clayton, Australia and were AR grade. All solvents were supplied by Ajax, Silverwater, Australia (AR grade).

### 4.2. Plant Source and Extraction

Pulp prepared from fresh *Terminalia ferdinandiana* fruit was provided by David Boehme (CEO of Northern Territory Wild Harvest, Australia). After pulping, the *T. ferdinandiana* fruit was frozen and maintained at −10 °C for transporting. A frozen voucher specimen (KP2014GD) is stored in the School of Environment and Science at Griffith University, Australia. The frozen pulp was thawed at room temperature and then dehydrated using a Sunbeam food dehydrator until a constant mass was recorded following additional drying time. The dried pulp was subsequently ground into a coarse powder using a coffee grinder and stored at −30 °C until use. For extraction, 1 g masses of ground fruit pulp powder was individually extracted by maceration in Falcon tubes containing either 50 mL of methanol, deionised water, ethyl acetate, chloroform or hexane. Each tube was extracted at 4 °C for 24 h with constant agitation on a shaker. Each extract was subsequently filtered through Whatman No. 54 filter paper to remove particulate matter. The organic solvents were removed by evaporation at room temperature in the shade, whilst the aqueous extract was dried by lyophilisation in a freeze dryer. The resultant dried extract pellets were then dissolved in a total of 10 mL of sterile deionised water containing 0.5% DMSO. The resuspended extracts were filtered using a 0.22 μm syringe-driven filter (Sarstedt, Mawson Lakes, Australia) and stored at 4 °C.

### 4.3. MS2 Phage Plaque Reduction Assay for Detection of Anti-RNA Viral Activity

The MS2 phage plaque reduction assay has been developed as a rapid and convenient preliminary method to screen for general anti-RNA virus activity [14]. The assay has previously been used effectively to screen plant extracts for antiviral activity to focus further on specific viral studies [14]. The MS2 bacteriophage and the F+ Amp+ *E. coli* strain used in this study were supplied by Dr. Jatinder Sidhu and Dr. Simon Toze of CSIRO, St. Lucia Qld, Australia. The MS2 plaque assay was performed as previously described [14]. Briefly, the methanolic, aqueous and chloroform extracts were adjusted to 5000 μg/mL. Due to lower extraction yields, the ethyl acetate and hexane extracts were adjusted to 2000 μg/mL in deionised water (containing 0.5% DMSO). A volume of 490 µL of each crude plant extract was inoculated individually with 10 μL of MS2 virus (containing approximately 1010 plaque forming units/mL) and incubated overnight at 4 °C. The solution was added to 500 μL F+ Amp+ *Escherichia coli* and incubated at 37 °C for 20 min. The bacteria/virus/extract mixture was then added to a 3 mL soft agar overlay (final concentration of 0.7% agar *w*/*v*) and immediately poured over pre-made agar plates (2.8% *w*/*v* agar). The plates were allowed to set for 15 min at room temp, and they were inverted and incubated overnight at 37 °C. The following day, the number of plaques were counted, and the percentage inhibition was recorded and compared to the negative control. Serial dilution was used to determine the antiviral strength of extracts and expressed as the concentration required to inhibit 50% of the plaque formation (IC_50_). Nutrient broth and deionised water were used as negative controls, whilst *Camellia sinensis* water extract (1000 µg/mL) and UV irradiation (microwave of 10 μL virus only for 4 × 30 s) were used as positive controls.

### 4.4. Inhibition of HIV-1 Reverse Transcriptase

#### 4.4.1. mRNA Synthesis and Extraction

To screen the extracts for the ability to inhibit HIV-1 reverse transcriptase (HIV-1 RT), the expression of the 18S rRNA gene was evaluated as a model system. HeLa cells (American Type Culture Collection, Rockville, MD, USA) were cultured and passaged in Roswell Park Memorial Institute (RPMI) 1640 medium (Invitrogen, Mt Waverley, Australia), containing 20 mm HEPES, 10 mm sodium bicarbonate, 50 IU/mL penicillin, 50 µg/mL streptomycin and 2 mm glutamine and supplemented with 10% foetal calf serum (Invitrogen, Australia, purchased from Thermo Fisher, Petaling Jaya, Australia). The HeLa cells were maintained in 75 mL flasks as monolayers in a 5% CO_2_ humidified atmosphere, incubated at 37 °C. Once the cells attained approximately 80% confluency, 1 mL of trypsin (Sigma Aldrich, Clayton, Australia) was added into the culture media, and the flask was re-incubated for 15 min in a 5% CO_2_ atmosphere at 37 °C to dislodge the HeLa cells from the flask surface. The media containing suspended HeLa cells suspensions were then transferred to a 10 mL centrifuge tube, and the cells were sedimented by centrifugation at 300× *g*. The supernatant was subsequently aspirated and discarded. The HeLa cell pellet was washed twice in 1 mL of PBS at pH 7.4. The final cellular pellet used for RNA extraction was resuspended in 500 µL of PBS at pH 7.4. Total RNA was extracted using RNeasy Mini Kits (Qiagen, Clayton, Australia) as per the manufacturer’s instructions, and total RNA yields were quantified by spectrophotometry using a Nanodrop (Thermo Fisher). The RNA was diluted to a concentration of 1 µg/µL in nuclease-free water and stored at −20 °C until use.

#### 4.4.2. Reverse Transcriptase Assay

The assay was performed using iScript cDNA synthesis kit (BioRad, Hercules, CA, USA) to perform the reverse transcriptase step as per the manufacturer’s instruction using random hexamer priming of the reactions. Reactions were performed in 20 µL volumes consisting of 4 µL 5× iScript reaction mix, 1 µL of 1 µg/µL total RNA extract and 1 µL reverse transcriptase (either Moloney murine leukaemia virus reverse transcriptase during the protocol development phase or HIV reverse transcriptase to determine anti-HIV-1 reverse transcriptase activity). Varying concentrations of *T. ferdinandiana* extracts were used for the final 14 µL of the reaction volume, with nuclease-free water used to make up the remaining volume to 20 µL. A negative control containing 1 μg of total RNA in nuclease-free water in addition to the standard reaction mix was included in the assay to quantify DNA synthesis in the absence of treatment. A growth control contained the methanolic *T. ferdinandiana* extract at 1000 μg/mL without the addition of total RNA template was also included to ensure no exogenous RNA was included in the assay via the test extracts. Vehicle controls consisted of the 0.5% DMSO in deionised water. Tannic acid (Sigma, Australia) was included in the assay as a positive control at a concentration of 10 μm, as it has previously been reported to be a good inhibitor of HIV reverse transcriptase [27,38]. The reverse transcriptase reactions were performed on a BioRad CFX96 thermocycler using the following reaction cycle: 5 min at 25 °C; 45 min at 37 °C; and 1 min at 95 °C. cDNA synthesis was assessed using qPCR amplification using the following primer set targeting the 18S rRNA gene: forward 5′-GTAACCCGTTGAACCCCATT-3′; reverse 5′-CCATCCAATCGGTAGTAGCG-3′ targeting a 150 base amplicon 18S ribosomal RNA gene (Genbank accession number: NR_146146). qPCR reactions were prepared using iQ SYBR Green Supermix (BioRad), 1 µL cDNA synthesis reaction mix, 1 µL primer mix containing 20 µm of both forward and reverse primers, 10 µL Supermix and 8 µL water. Quantitative PCR was performed using an initial enzymatic activation step consisting of holding the temperature at 95 °C for 3 min followed by 40 cycles of the following reaction steps, with fluorescent acquisition performed on the extension step: 95 °C for 15 s, 60 °C for 15 s and 72 °C for 30 s. Comparative quantitation was performed using the CFX Manager software (BioRad; version 2.1), and the fold change differences were compared to the positive control. The rate of reverse transcription in the presence of the *T. ferdinandiana* extracts (and positive control) were recorded across a range of concentrations, and linear regression was used to determine the concentration required to inhibit the enzyme activity by 50% (IC_50_).

### 4.5. Inhibition of HIV-1 Protease

HIV-1 protease inhibition was measured using a recombinant HIV-1 protease enzyme (Sigma, Australia; 100 Units/100 μg protein) and HIV protease substrate (500 μm; Sigma, Australia). The HIV protease substrate (Arg-Glu(EDANS)-Ser-Gln-Asn-Tyr-Pro-Ile-Val-Gln-Lys(DABCYL)-Arg) contains two modified amino acids on opposite sites of the Tyr-Pro cleavage site. When the substrate is cleaved, fluorescence is detected at an emission wavelength of 490 nm using an excitation wavelength of 340 nm. The substrate was prepared as a 100 μm stock solution in reaction buffer (0.1 M sodium acetate, 1 M sodium chloride, 1 mm EDTA, 1 mm DTT, 10% DMSO, 1 mg/mL BSA, Ph 4.7). A 20 μL aliquot of the substrate was added to 174 μL of the reaction buffer (in the presence or absence of the test extracts or controls) and dispensed into the wells of black flat-bottom Nunc-Immono 96 well plates. The reaction was initiated by the addition of 6 μL of HIV-1 protease enzyme, and the mixture was incubated for 1 h at 37 °C. Following the incubation, 20 μL of 5.5% trifluoroacetic acid (TFA) was added to quench the reaction. The fluorescence intensity was monitored at an emission wavelength of 490 nm on a fluorescence plate reader (ThermoFisher). Pepstatin A (Sigma, Australia) was included as a positive control at a concentration of 10 μm. The rates were recorded and calculated as a % of the untreated control using the following formula:

$$HIV\;protease\;activity\left(\%\;untreated\;control\right)=\left(\frac{A490\;ot\;the\;test}{A490\;of\;the\;untreated\;control}\right)*100$$

The reaction rates in the presence of the *T. ferdinandiana* extracts (and positive control) were recorded across a range of concentrations, and linear regression was used to determine the concentration required to inhibit the enzyme activity by 50% (IC_50_).

### 4.6. Inhibition of HIV-1 Infection in In Vitro Cell Assays

The ability of *T. ferdinandiana* extracts to inhibit HIV-1 infection was assessed in two in vitro assays. Both assays utilised HIV-1 pseudoviruses that are non-replicative and capable of only a single round of infection. The expression of HIV-1 tat-regulated firefly luciferase served as reporter for infection. For assessing the inhibition of viral entry, a well-established in vitro HIV-1 neutralisation assay [38] was used, with some modification. Pseudoviruses were generated through co-transfection of HEK293T cells with an env-deficient HIV-1 backbone vector (i.e., pSG3Δenv) and a rev/env expression vector (i.e., HIV-1 ConC or MLV) using polyethyleneimine (PEI, Polysciences, Warrington, PA, USA) to facilitate transduction. Pseudoviruses were harvested 48 h post-transfection and titered in TZM-bl cells. The vectors and TZM-bl cells were obtained from the NIH AIDS Reagent and Reference Program (Bethesda, MD, USA), while the HEK293T cells were obtained from the American Type Culture Collection (Innovation, VA, USA). Both the HEK283T and TZM-bl cell lines were maintained in complete Dulbecco’s Modified Eagle Medium (DMEM), consisting of high-glucose DMEM that was supplemented with 10% foetal bovine serum, 2 mm L-glutamine, 25 mm HEPES and 50 μg/mL gentamycin sulphate. Medium and supplements were purchased from Invitrogen (Carlsbad, CA, USA). For activity assessment, the extracts were dissolved in dimethyl sulfoxide (DMSO, Sigma-Aldrich, Taufkirchen, Germany) to 25 mg/mL and diluted to a 500 μg/mL working stock in complete DMEM. The working stock were added to duplicate wells of a 96-well culture plate (Sigma, Taufkirchen, Germany) and eight 2-fold serial dilutions (2–250 μg/mL final) were prepared in 50 μL complete DMEM per well. After the addition of 50 μL of pseudovirus to each well, the plate was incubated at 37 °C, 5% CO_2_ for 1 h, and 100 µL of TZM-bl cells at 1 × 10^5^ cells/mL was added. The plate was incubated for 48 h at 37 °C, 5% CO_2_.

For assessing the inhibition of early HIV-1 replication, a similar assay was employed, as previously described [39,40]. Pseudoviruses were generated through co-transfection of HEK293T cells with three vectors: pMD.G(29) to facilitate viral entry, pCSFLW for the expression of tat-regulated firefly luciferase, and the HIV-1 gag-pol expression vector p8.9MJ4. Pseudoviruses were harvested 48 h post-transfection and titered in HEK293T cells. The p8.9MJ4 and pMDG vectors were obtained from Didier Trono (École Polytechnique Fédérale de Lausanne, Lausanne, Switzerland), and pCSFLW was obtained from Nigel Temperton (University College London, London, United Kingdom). For activity assessment, the extracts were dissolved in dimethyl sulfoxide (DMSO, Sigma-Aldrich, Germany) to 25 mg/mL and diluted to a 500 μg/mL working stock in complete DMEM. The working stock was added to duplicate wells of a 96-well culture plate (Sigma, Germany), and eight 2-fold serial dilutions (2–250 μg/mL final) were prepared in 50 μL of complete DMEM per well. After the addition of HEK293T cells (at 4 × 10^5^ cells/mL) and pseudovirus to each well, the plate was incubated at 37 °C, 5% CO_2_, for 48 h.

For both assays, the expression of firefly luciferase was assessed with the Bright-Glo™ Luciferase Assay System (Promega, San Luis Obispo, CA, USA). Pseudoviral activity in the presence of *T. ferdinandiana* extracts was assessed relative to an untreated control, and the concentration required to inhibit 50% of viral *activity* (IC_50_) was determined from two independent screens in duplicate. The cytotoxicity of the extracts to TZM-bl and HEK293T cells was determined concurrently using the MTS-based CellTiter 96^®^ AQueous One Solution Cell Proliferation Assay (Promega, USA).

### 4.7. Toxicity Screening

The toxicity of the extracts was evaluated using two different assay methods. The *Artemia* nauplii lethality assay (ALA) provided rapid screening of the toxicity of the extracts. The cellular viability assay using MTS as a colorimetric indicator was used as a model to quantify the toxicity in mammalian cells.

#### 4.7.1. Evaluation of Toxicity by *Artemia franciscana* Nauplii Bioassays (ALA)

Potassium dichromate (K_2_Cr_2_O_7_) (AR grade, Chem-Supply, Gillman, Australia) was used as a positive control in the ALA. It was freshly prepared each day before use as a 1.6 mg/mL solution in sterile distilled water. The potassium dichromate solution was serially diluted in sterile artificial seawater and used for control toxin LC_50_ quantification studies in the ALA bioassay. Toxicity was evaluated using a modified ALA assay model described previously [15]. Following exposure to the extracts, negative control (artificial seawater) or toxin control for 24 h at 25 ± 1 °C, the wells were checked and the number of dead nauplii were counted. All treatments and controls were performed three times in triplicate (*n* = 9). The nauplii were then sacrificed by acidification of the seawater by the addition of 50 µL of glacial acetic acid (AR grade, Chem-Supply, Australia). The total number of nauplii were then counted and used to calculate the total % of mortality per well. Probit analysis with 95% confidence limits was used to calculate the LC_50_ with 95% value for each treatment.

#### 4.7.2. Human Dermal Fibroblast (HDF) Cell Viability Assay

Human primary dermal fibroblast (HDF) cell viability assays were also used to evaluate the toxicity of the *T. ferdinandiana* extracts. The HDF cells used in this study were purchased from American Type Culture Collection (ATCC PCS-201-012). The cells were cultured in Dulbecco’s Modified Eagle Medium (DMEM; ThermoFisher Scientific, Petaling Jaya, Australia), supplemented with, 50 µg/mL streptomycin and 50 IU/mL penicillin (Sigma Aldrich, Australia), as well as 10% foetal calf serum (Invitrogen, Australia). The cells were cultured and maintained in a 5% CO_2_ humidified atmosphere at 37 °C, in 75 mL flasks until the monolayers reached ~80% confluency. Then, 1 mL of trypsin (Sigma Aldrich, Australia) was added into the culture medium, and the flasks were reincubated in 5% CO_2_ at 37 °C for a further 15 min to dislodge the HDF cells from the monolayer. The resultant cell suspensions were subsequently sedimented by centrifugation in sterile 10 mL centrifuge tubes (Sarstedt, Australia). The supernatants were aspirated and discarded, whilst the cells were resuspended in 9 mL of fresh culture media. Then, 70 µL volumes of the resuspended cells (containing ~5000 cells) were dispensed to all of the wells on a sterile, flat-bottomed 96-well plate. Next, 30 µL volumes of the test extracts or cell media (for the negative control) were individually added to wells, and the cells were re-incubated for 24 h in a humidified 5% CO_2_ atmosphere at 37 °C. The extracts were all screened at a concentration of 300 µg/mL. The cells were then washed in phosphate-buffered saline solution (PBS; pH 7.2); then, 20 µL of Cell Titre 96 Aqueous One solution (Promega, Sydney, Australia) was added to each well as an indicator reagent. The plates were then incubated for 3 h to allow for colour development. Cell viability was quantified by measuring the absorbances (test wavelength = 540 nm, blank wavelength = 690 nm) using a Molecular Devices Spectra Max M3 plate reader (Molecular Devices, San Jose, CA, USA). All tests were performed in triplicate, each test with three internal replicates. The cellular viability (%) of each well was calculated using the following formula:

$$\%\;cellular\;viability=\frac{Abs\;test\;sample-(mean\;Abs\;control-mean\;Abs\;blank)}{\left(mean\;Abs\;control-mean\;Abs\;blank\right)}$$

Wells with cellular viability ≤50% compared to the untreated control (untreated cells) indicated toxicity. In contrast, wells with >50% cell viability (compared to the untreated control) were deemed to be nontoxic.

#### 4.7.3. Therapeutic Index Evaluation

The therapeutic index of each test was calculated using the following formula as a measure of their suitability as therapeutic targets:

Therapeutic index = (ALA LC_50_)/(the relevant IC_50_)

### 4.8. Non-Targeted Metabolomic HPLC-MS QTOF Profile Analysis

Non-targeted metabolomic profiling analysis was undertaken using standard methods developed by our group [41]. Briefly, a 2 µL volume of test extract was separated on an Agilent 1290 HPLC system equipped with a Zorbax Eclipse plus C18 column (2.1 × 100 mm, 1.8 µm particle size) (Agilent Technologies, Santa Clara, CA, USA). The chromatography utilised a gradient system that consisted of two solvents: (A) ultrapure water, and (B) 95:5 acetonitrile/water. The mobile phases were modified by the addition of 0.1% (*v*/*v*) glacial acetic acid for mass spectrometry (MS). Analysis of the eluted compounds was run in positive mode, or with 5 mm ammonium acetate for MS analysis in negative ion analysis mode. Chromatograms were run at a flow rate of 0.7 mL/min, using the following gradient conditions: 5 min isocratic elution at 5% B, followed by a gradient from 5% to 100% mobile phase B over a period of 25 min. Mobile phase B was then maintained at 100% for a 3 min isocratic elution. An Agilent 6530 quadrupole time-of-flight spectrometer, fitted with a Jetstream electrospray ionisation source, was used for mass spectrometry analysis in both positive and negative mode. The data obtained were subsequently analysed using the Masshunter Qualitative analysis software package (B08.00; Agilent Technologies, Santa Clara, CA, USA). The software’s Find by Molecular Feature function was used to generate a putative list of compounds for each extract. Each of the generated compound lists were compared with two accurate mass databases, including a database developed for this study (which contained 800 compounds), as well as the Metlin metabolomics database (24,768 compounds). The Find Formula function of the software was then used to generate empirical formulae for any unidentified compounds.

### 4.9. Statistical Analysis

All bioassay data reported herein are expressed as the mean ± SEM of three independent experiments, each with internal triplicates (*n* = 9) unless otherwise stated. One-way analysis of variance (ANOVA) was used to calculate statistical significance between the negative control and treated groups. *p* values < 0.01 were considered to be statistically significant.

## 5. Conclusions

This study reports that methanolic and aqueous *T. ferdinandiana* extracts have potent anti-HIV-1 activity via inhibition of at least three phases of the HIV-1 replicative cycle. The extracts are potent inhibitors of viral entry, HIV-1 reverse transcriptase and HIV-1 protease. LC-MS analysis identified and highlighted several tannins, flavonoids and stilbenes in the methanolic extract. All of these compounds have previously been reported to either have anti-HIV-1 activities or to have activities consistent with the inhibition of one or more phases of HIV-1 replication. However, these compounds were putatively identified by metabolomic profiling techniques, and future studies are required to isolate these compounds and rigorously verify their identity. Additionally, future studies should combine quantitative LC-MS/MS studies with bioactivity-driven separation studies to verify the identity of the compounds that contribute to the bioactivities reported in our study. Whilst the effect of the extracts on HIV-1 integrase activity was not tested in our study, several of the identified tannins and flavonoids have previously been reported to have potent HIV-1 integrase activity. It is therefore likely that future testing may also detect inhibition of the HIV-1 integrase. Furthermore, the extracts were nontoxic or of low toxicity, resulting in clinically relevant therapeutic indexes. Based on their potent inhibition of HIV-1 cell entry, reverse transcriptase and protease enzymatic activities and the safety profiles of the extracts, we conclude that the *T. ferdinandiana* methanolic and aqueous extracts represent promising new herbal therapies for the prevention, treatment and management of HIV.

## Figures and Tables

**Table 1:** IC_50_ (µg/mL) values of *T. ferdinandiana* fruit extracts against MS2 phage and various phases of HIV-1 replication, as well as the detection and quantification of toxicity LC_50_ values (µg/mL).

		Methanol Extract			Water Extract			Therapeutic Index					
		MS2	HIV	MLV	MS2	HIV	MLV	MS2		HIV		MLV	
								M	W	M	W	M	W
**Antiviral Assays**	**MS2 Viral replication**	68	ND	ND	44	ND	ND	13	22	ND	ND	ND	ND
	**Reverse transcriptase**	ND	35	ND	ND	33	ND	ND	ND	13	19	ND	ND
	**Protease**	ND	19	ND	ND	27	ND	ND	ND	46	36	ND	ND
	**Cell entry**	ND	16	9	ND	19	19	ND	ND	15	13	25	13
	**Early phase replication in HEK293T cells**	ND	171	ND	ND	138	ND	ND	ND	0.7	1	ND	ND
**Toxicity**	**ALA toxicity**	875			983								
	**HDF toxicity**	-			-								
	**TZM-bl toxicity**	227			>250								
	**HEK293T toxicity**	112			138								

Values indicate mean IC_50_ or LC_50_ values. M = methanolic extract; W = water extract; MLV = murine leukaemia virus; ND indicates that an IC_50_ or LC_50_ value was not determined;—indicates that the extract was not toxic as cellular viability was >50% of the untreated control cell viability. The therapeutic index of the MS2 phage and HIV-1 enzyme assays were calculated using the ALA toxicity values, whilst the therapeutic index for the cell line studies was calculated using the toxicities in the individual cell lines.

**Table 2:** Qualitative HPLC-MS/MS analysis of the *T. ferdinandiana* leaf extracts, elucidation of empirical formulas and identification (where possible) of the compound.

Putative Identification	Empirical Formula	Retention Time	Molecular Mass	Relative Abundance (% Total Peak Area)	
				Positive Ionisation Mode	Negative Ionisation Mode
Diethylstilbestrol monosulfate	C_18_H_20_O_5_S	1.805	348.1036	0.12	<0.1
Protocatechuic acid	C_7_H_6_O_4_	0.522	154.0272	<0.1	2.34
Ellagic acid dihydrate	C_14_H_10_O_10_	1.085	338.0281	0.15	<0.1
Gallic acid	C_4_H_8_O_5_	1.383	136.0374	4.3	4.7
Chebulic acid	C_14_H_12_O_11_	1.54	356.039	0.67	0.61
Pyrocatechol glucronide	C_12_H_14_O_8_	1.84	286.069	0.22	<0.1
Ethyl gallate isomer 1	C_9_H_10_O_5_	2.33	198.0529	0.04	<0.1
Chlorogenic acid	C_16_H_18_O_9_	3.52	354.311	0.86	<0.1
Ellagic acid	C_14_H_6_O_8_	4.277	285.1943	0.56	0.83
Ethyl gallate isomer 2	C_9_H_10_O_5_	4.871	198.0529	0.14	<0.1
Corilagin	C_27_H_22_O_18_	4.943	634.082	<0.1	2.83
Ethyl gallate isomer 3	C_9_H_10_O_5_	6.065	198.0529	0.17	1.24
Gallocatechin	C_15_H_14_O_7_	6.142	306.0768	<0.1	<0.1
Resveratrol	C_14_H_12_O_3_	6.235	228.2433	0.75	<0.1
Resveratrol glucoside	C_20_ H_22_ O_8_	6.924	390.1333	0.11	<0.1
Combretastastin A-1	C_18_H_20_O_6_	6.94	332.1245	0.1	<0.1
Combretastastin	C_18_H_22_O_6_	7.274	334.1403	0.16	<0.1
Pentahydroxystilbene	C_14_H_12_O_5_	8.322	260.068	0.11	<0.1
Luteolin	C_21_H_20_O_11_	8.345	448.1025	0.54	<0.1
Castalagin	C_41_H_26_O_26_	7.015	934.0715	0.12	<0.1
Rutin	C_27_H_30_O_16_	8.73	610.521	0.19	<0.1
Chebulinic acid	C_41_H_32_O_27_	8.996	956.113	0.67	<0.1
Exifone	C_13_H_10_O_7_	9.32	278.0433	1.62	2.26
Punicalin	C_34_H_22_O_22_	9.363	782.0621	0.165	<0.1
Vitexin	C_21_H_20_O_10_	9.397	432.1064	5.59	0.77
Chebulagic acid	C_41_H_30_O_27_	9.878	954.0965	<0.1	0.13
Quercetin	C_15_H_10_O_7_	11.293	302.0434	1.03	0.23
Trimethyl ellagic acid	C_17_H_12_O_8_	14.237	344.0538	0.17	1.07

% peak area refers to the peak area for the individual compound expressed as a % of the total area under peaks for the chromatogram in the relevant mode.

## Data Availability

Data are available from the corresponding author upon reasonable request.
